# Polyclonal carbapenemase-producing *Escherichia coli* in Northern Italy: the emergence of NDM-7

**DOI:** 10.3389/fcimb.2025.1519827

**Published:** 2025-03-14

**Authors:** Vittoria Mattioni Marchetti, Marta Corbella, Aurora Piazza, Stefano Gaiarsa, Irene Mileto, Cristina Merla, Angela Kuka, Aseel AbuAlshaar, Patrizia Cambieri, Roberta Migliavacca, Fausto Baldanti

**Affiliations:** ^1^ Department of Clinical, Surgical, Diagnostic and Paediatric Sciences, University of Pavia, Pavia, Italy; ^2^ Specialization School of Microbiology and Virology, University of Pavia, Pavia, Italy; ^3^ Microbiology and Virology Unit, IRCCS Fondazione Policlinico San Matteo, Pavia, Italy; ^4^ IRCCS Fondazione Policlinico San Matteo, Pavia, Italy; ^5^ Department of Biology and Biotechnology, American University of Madaba, Madaba, Jordan

**Keywords:** NDM-7, *Escherichia coli*, carbapenemases, IncX3, WGS, ST401, ST355, virulence

## Abstract

The spread of extended-spectrum beta-lactamase (ESBL)- and carbapenemase-producing *Escherichia coli* clones in humans, animals, and the environment is of great concern worldwide. In this study, we characterized four carbapenemase-producing *E. coli* (CP-Ec) isolated from human samples. Two isolates of ST401, rarely associated with carbapenemase and/or ESBL resistance genes, harbored *bla*KPC-3 and *bla*VIM-1 genes, respectively, and were genetically distant from each other. One CP-Ec isolate belonging to ST355, typically found in poultry and environmental sources and not associated with carbapenemases, was *bla*KPC-3 positive and showed a wide range of virulence genes. The last CP-Ec strain belonged to ST3564, previously described in livestock with a large virulome but no carbapenemase. The CP-Ec ST3564 isolate co-harbored *bla*VIM-1 and *bla*NDM-7 genes, which, to our knowledge, have not been previously reported in Italy. These results emphasize the crucial role of a genomic-based surveillance program to intercept the emergence of critical *E. coli* clones.

## Introduction

*Escherichia coli* is a natural inhabitant of and the most common bacterial species colonizing the gastrointestinal tract of warm-blooded animals, including humans. It is classified as a Gram-negative commensal bacterium and facultative pathogen, since it is an important cause of morbidity and mortality in humans and animals worldwide, associated with both subclinical disorders and a wide range of clinical conditions, including enteric, urinary, and systemic infections (García and Fox, 2021). *E. coli* can also be considered a potential zoonotic pathogen, being ubiquitous in most food animals and their environment, including wastewater and vegetable products. The exceptionally versatile genome of the members of the *E. coli* genus reflects their multifaceted nature, supported by the presence of several different strains showing diverse combinations of resistance genes and virulence factors. Depending on the virulence factors encoded and expressed, *E. coli* strains are classified into specific pathotypes. There are two main classifications of pathogenic *E. coli*: diarrheagenic *E. coli* (DEC) and extraintestinal pathogenic *E. coli* (ExPEC). Among DEC, there are at least nine well-recognized pathotypes: enterotoxigenic *E. coli* (ETEC), enteropathogenic *E. coli* (EPEC), enterohemorrhagic *E. coli* (EHEC), enteroaggregative *E. coli* (EAEC), Shiga toxin (Stx)-producing *E. coli* (STEC), enteroinvasive *E. coli* (EIEC), diffusely adhering *E. coli* (DAEC), adherent-invasive *E. coli* (AIEC), and cell-detaching *E. coli* (CDEC) (Braz et al., 2020). All these pathotypes are common among both humans and animals.

ExPEC can cause a wide variety of infections, and the main pathotypes among this group are uropathogenic *E. coli* (UPEC), sepsis-causing *E. coli* (SEPEC), and neonatal meningitis-associated *E. coli* (NMEC). Following a One Health perspective, the avian pathogenic *E. coli* (APEC) has also been included in the ExPEC group causing diverse local and systemic infections in poultry, such as chickens, turkeys, ducks, and many other avian species. APEC are now recognized as foodborne zoonotic pathogens as well as a source or reservoir of extraintestinal infections in humans (Kathayat et al., 2021).

Antimicrobial resistance (AMR), a global issue contributing to untreatable infections, is driven by antibiotic exposure in healthcare, agriculture (animals, plants, or food-processing technology), and the environment (sea, soil, drinking water, and wastewater) (Smit et al., 2023). Extended-spectrum beta-lactamase (ESBL)- and carbapenemase-producing *E. coli* are of great concern worldwide, considering also that both resistance genes and virulence traits can be carried on chromosome and on mobile genetic elements, as plasmids, the latter being transferable intra- and inter-species. In addition to third-generation cephalosporin and carbapenem resistance, fluoroquinolone and aminoglycoside resistance is also particularly worrisome in *E. coli* (Kocsis et al., 2022). The dissemination of these elements, and the convergence of wide resistomes and virulomes, is associated with and contributed to the successful expansion of international high-risk clones of critical priority, often shared between different hosts and environments (Fuga et al., 2022). Many of the high-risk *E. coli* pandemic clones are classified into eight phylogroups (A, B1, B2, C, D, E, F, and G) and in different sequence types (STs). This classification of *E. coli*, together with the analysis of virulence and resistance traits, has been very useful to investigating the distribution of *E. coli* clones among hospital, community, animal and environment settings. It has been suggested that group B2 strains may be specific to humans and that some group B1 strains harboring the *hly* gene may be specific to animals (Clermont et al., 2011). Phylogroup A, considered one of the most ancestral groups, is primarily associated with commensal, non-pathogenic strains and commonly found in the environment. Phylogroup C strains of *E. coli* are less common; they can be found in both humans and animals, even though evidence of their association with specific human diseases is scarce. The boundary between settings has long been crossed, and it is increasingly frequent to identify *E. coli* clones, previously typically of one, causing epidemics in others. Nowadays, some multidrug-resistant (MDR) high-risk clones, such as ST131, ST10, ST69, ST73, ST405, ST410, and ST457, have been established, but several less frequent clones overlapping human and non-human sources are emerging.

This study aims to characterize at the genomic level four carbapenem-resistant *E. coli* (CR-Ec) isolated from human samples, collected at the Fondazione IRCCS Policlinico San Matteo hospital in Pavia (Italy), to evaluate their resistance, virulence traits, and association with high-risk clones.

## Materials and methods

### Bacterial identification and antimicrobial susceptibility testing

The four strains of this study were isolated from the rectal swabs of four patients admitted to the Intensive Care Unit and the Hematology and the General Surgery wards of the Fondazione IRCCS Policlinico San Matteo hospital in Pavia (Italy) from November 2022 to September 2023.

Rectal swabs were plated on chromogenic medium for carbapenemase-producing *Enterobacteriaceae*, CHROMID Carba (bioMérieux, Marcy-l’Étoile, France). Species identification was performed by matrix-assisted laser desorption ionization time-of-flight mass spectrometry (MALDI-TOF MS) (Bruker Daltonics GmbH) and analyzed using BioTyper version 3.0. Carbapenemase production was tested using the NG-Test^®^ CARBA-5 immunoassay (NG Biotech Laboratories, Guipry, France). Antimicrobial susceptibility testing (AST) was carried out according to a standard internal laboratory protocol using Thermo Scientific™ Sensititre™ Gram Negative MIC Plates DKMGN. The minimum inhibitory concentration (MIC) values for fosfomycin were obtained using the agar dilution kit (Liofilchem, Roseto Degli Abruzzi, Teramo, Italy). MIC values were interpreted according to the European Committee on Antimicrobial Susceptibility Testing (EUCAST) version 13 breakpoints (EUCAST breakpoint tables for interpretation of MICs and zone diameters: http://www.eucast.org).

### Short-read and long-read whole-genome sequencing

The four strains underwent whole-genome sequencing (WGS). The genomic DNA, extracted using the DNeasy Blood & Tissue Kit (Qiagen, Hilden, Germany), was sequenced on an Illumina MiSeq platform (Illumina Inc., San Diego, CA, USA) with 250-bp paired-end sequencing, after library preparation with the Nextera XT library preparation kit. Reads were quality-checked with FastQC v0.11.9 (https://www.bioinformatics.babraham.ac.uk/projects/fastqc). To investigate plasmid sequence content and structure, all four CR-Ec strains were also sequenced using the Oxford Nanopore technology: long-read sequencing was performed with a Flongle R10 flow cell, after library preparation with Rapid Barcoding Kit 96 V14. Assemblies were annotated using Prokka and manually checked by the Rapid Annotation using Subsystems Technology (RAST) server. The resistome, plasmid replicon content, and virulome were determined using ResFinder 4.1, PlasmidFinder 2.1, and the Virulence Factor Database (VFDB) via ABRicate, respectively. Multilocus sequence typing (MLST) was carried out through the *E. coli* Warwick scheme via the EnteroBase website (https://enterobase.warwick.ac.uk/). Genomic information about both types of sequencing is reported in **Supplementary Table 2**.

### SNP analysis and phylogenetic reconstruction

The phylogenetic relationships between the four studied strains and global genomes were investigated. Specific datasets for each ST were built by downloading available genomes from EnteroBase (https://enterobase.warwick.ac.uk/). In detail, a total of 172 public *E. coli* genomes were retrieved for ST401, 409 genomes for ST355, and 12 for ST3564, including both complete and draft genomes. Single-nucleotide polymorphism (SNP)-based phylogenies were obtained using parsnp v1.2 (https://github.com/marbl/parsnp/), and evolutionarily related high-quality genomes were used as references. Graphic illustrations of the trees were built with Interactive Tree of Life (iTOL) (https://itol.embl.de/). Pairwise SNPs between strains were counted from the core SNP alignment with snp-dists 0.7.0 (https://github.com/tseemann/snp-dists).

### Conjugation/transformation assay

The transferability of carbapenemases genes was tested in liquid medium using the *E. coli* J62 strain (pro-, his-, trp-, lac-, Sm^R^) as recipient. The four *E. coli* clinical isolates, the donor strains, in the logarithmic phase of growth were mixed with recipients in the early stationary phase in a 1:10 ratio in Mueller Hinton broth, and the mixture was incubated at 37°C overnight. Transconjugants were selected on MacConkey agar plates (Scharlab, SL, Barcelona, Spain) containing streptomycin (1,000 mg/L) (Sigma-Aldrich, St. Louis, MO, USA) and cefotaxime (16 mg/L) (Sigma-Aldrich). Plasmid transferability was confirmed for CR-Ec 9008 and CR-Ec 8501 through NG-CARBA5 and PCR replicon typing (PBRT 2.0 kit, Diatheva), respectively.

### Plasmids’ visualization

BRIG v.0.95 was used to produce figures of comparison of the circular plasmids’ sequences. A linear map of pQil plasmid environments was created by using Easyfig (Sullivan et al., 2011) and the graphic editor Inkscape (https://inkscape.org/it/).

### Data availability

The nucleotide sequences of the four genomes were deposited and are available in the National Center for Biotechnology Information (NCBI) Genome database under BioProject ID PRJNA1166141.

## Results

From November 2022 to September 2023, four CR-Ec strains were isolated from the rectal swabs of as many patients and sequenced. All four isolates, named 7521, 7926, 8501, and 9008, were resistant to aztreonam, amoxicillin/clavulanate, cephalosporins, ertapenem, and ceftolozane/tazobactam and susceptible to colistin and fosfomycin (**Supplementary Table 1**). Resistance to a higher number of antibiotics was observed for the two CR-Ec strains 8501 and 9008, being resistant also to ceftazidime/avibactam, imipenem, imipenem/relebactam, meropenem, meropenem/vaborbactam, gentamicin, and trimethoprim/sulfamethoxazole. The immunochromatographic assay detected the production of *Klebsiella pneumoniae* carbapenemase (KPC) for strains 7521 and 7926, VIM and NDM for 8501, and VIM for 9008.

**Table 1** shows the results of the WGS analyses. *In silico* MLST identified ST401 for both CR-Ec 9008 and CR-Ec 7521, ST355 for CR-Ec 7926, and ST3564 for CR-Ec 8501. The four studied strains revealed a heterogeneous phenotype: CR-Ec 9008 and CR-Ec 7521 belonged to phylogroup A and serotypes O21:H33 and O15:H11, respectively; CR-Ec 7926 to phylogroup B2 and serotype O1:H5; and CR-Ec 8501 to phylogroup C and serotype O8:H19 (**Table 1**).

**Table 1:** Metadata and molecular results of the four studied *E. coli* strains.

ID	Isolation date	Ward	Serotype, phylogroup	ST	Aminoglycosides	β-Lactams	Phenicols	Trimethroprim	Macrolides	Quinolones	Sulfonamides	Tetracyclines	Plasmids
7521	12.11.2022	General Surgery	O15:H11, A	ST401	ND*	*bla*KPC-3, *bla*OXA-9	ND*	ND*	ND*	ND*	ND*	ND*	ColRNAI, IncFIB(pQil), IncFII, IncX5
7926	17.02.2023	Intensive Care Unit	O1:H5, B2	ST355	ND*	*bla*KPC-3	ND*	ND*	ND*	ND*	ND*	ND*	Col(MG828), Col156, IncFIB(AP001918), IncFIB(pQil), IncFII
8501	23.06.2023	Intensive Care Unit	O8:H19, C	ST3564	*aac(6*′*)-Ib*, *aadA1*, *aph(3*′*)-XV*, *aph(6)-Id*	*bla*CTX-M-15, *bla*NDM-7, *bla*OXA-1, *bla*VIM-1, *ble*-MBL	ND*	*dfrA14*	ND*	*qnrB2*	*sul1, sul2*	*tet(A)*	IncFIB(K), IncFII, IncN, IncX3
9008	25.09.2023	Hematology	O21:H33, A	ST401	*aac(3)-IIe*, *aph(3*″*)-Ib*	*bla*SHV-12, *bla*VIM-1	*catB2*	*dfrA14*	*mph(A)*	*qnrS1*	*sul1*	ND*	Col(Ye4449), ColRNAI, IncA/C, IncFIA(HI1), IncFIB(K), IncFII

*ND, not detected.

Concerning carbapenem resistance genes, CR-Ec 7926 and CR-Ec 7521 carried *bla*KPC-3, CR-Ec 9008 carried *bla*VIM-1, and CR-Ec 8501 co-harbored *bla*NDM-7 and *bla*VIM-1. Based on the short and long sequencing, the four strains had a heterogeneous plasmidome. CR-Ec 7521 contained an IncFIB(pQiL)-IncFII(k) plasmid of 107,213 bp, an IncX5 plasmid of 39,317 bp, and a p0111 plasmid of 57,904 bp; CR-Ec 7926 showed an IncFII-IncFIB(pQiL)-IncFII(k) plasmid of 87,125 bp, an IncFIA-IncFIB(AP001918) of 114,034 bp, and a small Col156 plasmid of 6,646 bp. Differently, CR-Ec 8501 carried an IncFIB(k)-IncFII(k) plasmid of 175,363 bp, an IncN plasmid of 54,469 bp, and an IncX_3_ plasmid of 45,122 bp; while CR-Ec 9008 included an IncFIA(HI1)-IncFIB(k) plasmid of 154,767 bp and an IncA plasmid of 152,625 bp.

### *E. coli* ST401

**Figure 1:**
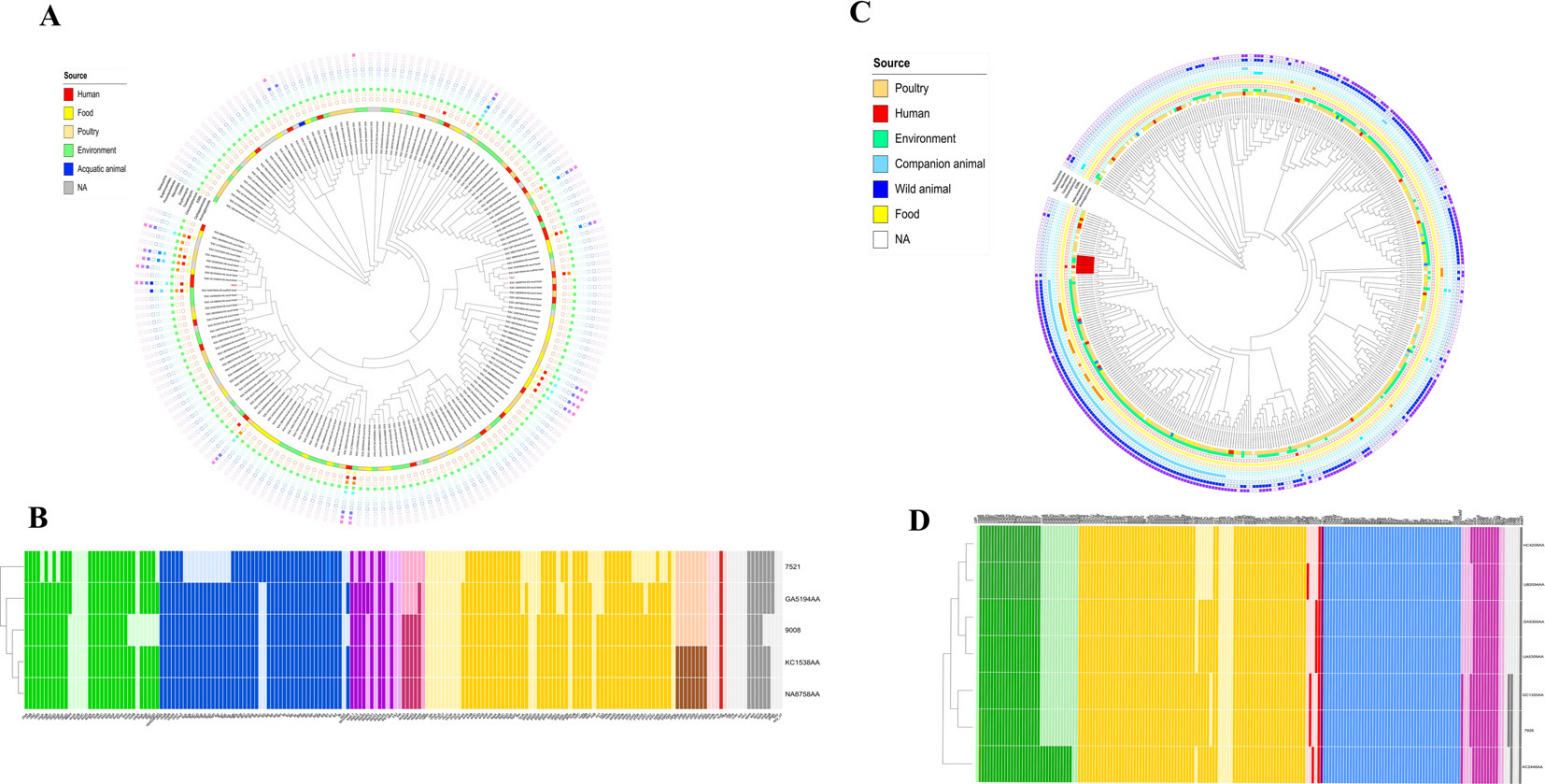
**(A)** iTOL V6 representation of the SNP-based tree for the 173 genomes of *E. coli* ST401 obtained by parsnp and related resistome. Yellow grid = aminoglycoside resistance genes; red grid = carbapenem resistance genes; orange grid = ESBL genes; green grid = chloramphenicol resistance genes; cadet blue grid = trimethoprim resistance genes; aqua grid = erythromycin resistance genes; police strobe grid = colistin resistance genes; ocean blue grid = macrolides resistance genes; soft purple grid = fluoroquinolone resistance genes; wisteria grid = sulfonamide resistance genes; pink purple grid = tetracycline resistance genes. **(B)** Heatmap representation of the virulence gene content. Colors describe virulence genes involved in adhesion (green), chemotaxis (blue), invasion (purple), effector system (pink), metabolism (yellow), siderophores (brown), toxins (red), and others (gray). **(C)** iTOL V6 representation of the SNP-based tree for the 410 genomes of *E. coli* ST355 obtained by parsnp and related resistome. Seafoam grid = aminoglycoside resistance genes; red grid = carbapenem resistance genes; yellow grid = beta-lactamase genes; orange grid = ESBL genes; cadet blue grid = chloramphenicol resistance genes; sky blue grid = trimethoprim resistance genes; steel blue grid = macrolides resistance genes; azure grid = sulfonamide resistance genes; purple grid = tetracycline resistance genes. **(D)** Heatmap representation of the virulence gene content. Colors describe virulence genes involved in adhesion (green), metabolism (yellow), toxins (red), effector delivery system (purple), chemotaxis (light blue), effector system (pink), siderophores (brown), and others (gray).

The SNP-based phylogeny of CR-Ec 9008 and 7521 against all of the 172 *E. coli* ST401 genomes available in the EnteroBase database showed that CR-Ec 9008 had the highest relatedness with genome KC1538AA (number of SNPs = 55) (collected from a human source in Canada) and genome NA8758AA (number of SNPs = 56) (from environmental sampling in the UK), while CR-Ec 7521 had the highest relatedness with KC8458AA (number of SNPs = 691), collected in Kenya in 2016 (**Figure 1A**). Strains 9008 and 7521 turned out to be evolutionarily distant (number of SNPs = 4,825) and fell in two different clusters. Considering the AMR gene content, ST401 is rarely associated with carbapenem resistance genes, found in 12 cases among the 172 available genomes, as well as with ESBL genes (*bla*CTX-M types), detected in only 6 cases (**Figure 1A**). Strains 9008 and 7521 had a slightly different virulome, which included genes for adhesion (such as *csgA*, *fdeC*, *fimH*, *lpfA*, and *nlpl*), metabolism (*gad* and *terC*), and invasion (*clpK1-2*, *traT*, and *yehABCD*). Interestingly, both strains harbored the avian toxin *hlyE* genes (**Figure 1B**).

### *E. coli* ST355

The SNP-based phylogeny of strain 7926 and all of the *E. coli* ST355 genomes (n = 409) available on the EnteroBase database showed a genomic link between CR-Ec 7926 and a small cluster of genomes from both poultry and environmental sources. In detail, the highest similarity (number of SNPs = 80) was assessed within CR-Ec 7926 and GC1320AA, collected in 2022 in the USA from poultry. Moreover, these genomes clustered together (number of SNPs > 180) with GA5300AA (collected in 2015), HC4209AA (collected on 2022 in the USA from poultry), AC2446AA (collected in 2018 from New Zealand soil), UA5309AA (collected in 2017 in the USA from poultry), and UB2094AA (obtained in 2017 from Japanese lake water) (**Figure 1C**). While the SNP count remains high, these data suggest a temporary evolution of a small cluster of ST355, attesting its emergence in the human host. Based on the available metadata, *E. coli* ST355 turned out to be not associated with carbapenemase genes, while resistance to aminoglycosides, sulfonamides, and tetracycline is common.

Concerning the virulome, CR-Ec 7926 had a wide range of virulence genes, including those implicated in adhesion (*csg* locus, *ecp* locus, and *fim* locus), metabolism (*chuA*, *ent*, *fep*, and *gad* loci), invasion (*traT* and *ibeABC*), and toxins (*vat*) (**Figure 1D**). Compared with closely related strains, CR-Ec 7926 consisted of a similar virulome content, except for the toxin *ccdb* gene, responsible for the post-segregational killing of plasmid-free cells, shared with GC1320AA only. Moreover, all the seven compared genomes harbored the vacuolating autotransporter toxin *vat* gene, involved in cystitis and pyelonephritis by UPEC.

### Genomic composition of *bla*KPC-3-harboring pQil plasmids

CR-Ec 7521 and 7926 shared a pQil plasmid (query 81%, identity 99.99%), belonging to IncFIIk, of different sizes: 107,213 bp and 87,125 bp, respectively. Both plasmids have in common genes for type I R-M systems, catalyzing restriction and modification activity; the *tra* locus (34,158 bp), implicated in the transferability process; the *parAB* partition system; and the *bla*KPC-3 transposon. For both plasmids, *bla*KPC-3 was carried on Tn*4401b*, showing no deletions upstream *bla*KPC-3 gene (**Supplementary Figure 1**). Additionally, 7521-pQil and 7926 shared, with opposite orientations, a 10,319-bp region enriched in stabilization genes. In detail, this region had *parM* involved in a plasmid partitioning system, antirestriction genes (*ardA* and *klcA*) regulating gene transferability, DNA methyltransferase, and *umuC* and *umuD* genes for the DNA repairing system. 7521-pQil had a further region (6,108 bp) carrying two copies of *bla*OXA-9, flanked by InsA-InsB and Tn*5403*. Moreover, 7521-pQil had an additional region of 12,688 bp containing genes for plasmid stabilization and regulation: single-strand binding DNA genes (*ssb*) implicated in the maintenance of DNA metabolism during replication/repair/recombination phases and *psiA*-*psiB* and *parM* involved in the plasmid partitioning system and DNA methyltransferase (**Supplementary Figure 1**).

### *E. coli* ST3564

**Figure 2:**
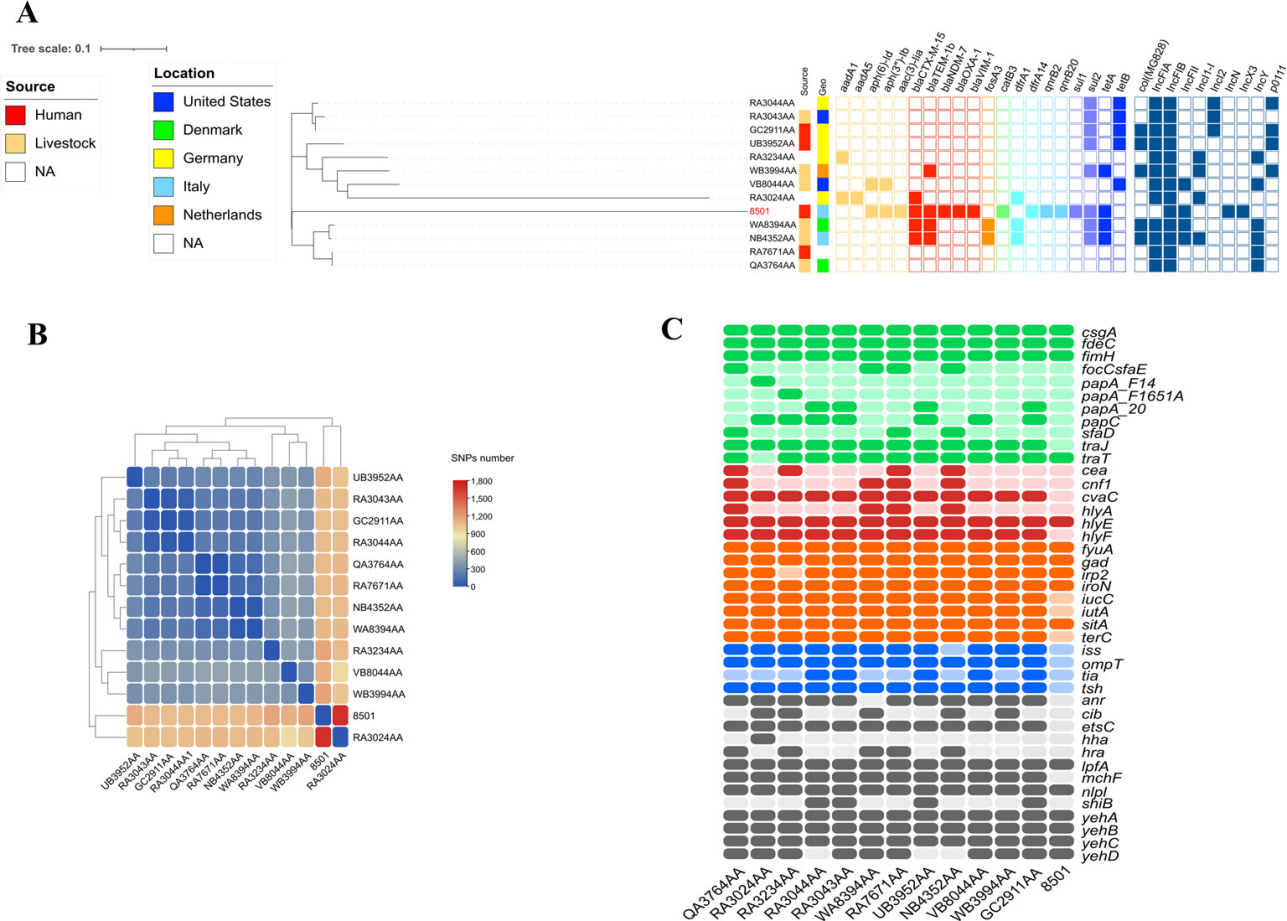
**(A)** Heatmap representation of SNPs number within the 13 genomes of *E. coli* ST3564. **(B)** iTOL V6 representation of SNP-based tree for the 410 genomes of *E. coli* ST355 obtained by parsnp, related resistome, and plasmidome. Yellow grid = aminoglycoside resistance genes; red grid = beta-lactamase resistance genes; orange grid = fosfomycin resistance genes; light green grid = chloramphenicol resistance genes; cadet blue grid = trimethoprim resistance genes; lilac grid = sulfonamide resistance genes; navy blue grid = tetracycline resistance genes; steel blue grid = plasmid content. **(C)** Heatmap representation of the virulence gene content. Colors describe virulence genes involved in adhesion (green), toxins (red), metabolism (orange), invasion (blue), and others (gray).

Strain 8501 belonged to ST3564 (clonal complex 23) and showed a different evolution path. Compared with the 12 genomes available on the EnteroBase, CR-Ec 8501 turned out to be phylogenetically distant (up to 1,705 SNPs detected) (**Figures 2A, B**). The closest isolate (number of SNPs = 1,053) was GC2911AA, collected in Germany in 2015 from an animal source. GC2911AA showed a resistome composed of genes for sulfonamides (*sul2*) and tetracycline (*tet(B)*) only and a plasmidome of Col(MG828), IncFIA, IncFIB(AP001918), IncI2(Delta), and p0111 (**Figure 2B**). The 12 genomes of ST3564 showed an overall narrow resistome, mainly composed of sulfonamides (*sul*-like), tetracycline (*tet*-like), and rarely β-lactams (*bla*CTX-M-like) and fosfomycin (*fosA3*) resistance genes. Considering the virulence gene content, the 12 genomes showed a wide virulome, characterized by several toxin genes (*cea*, *cnf1*, *hlyA*, *hlyE*, and *hlyF*). Differently, CR-Ec 8501 possessed a limited virulome, including *hlyE* as toxin gene (**Figure 2C**). The decreased virulence power is compensated for with an enlarged resistome for 8501, including aminoglycosides (*aph(6)-Id*, *aph(3*″*)-Ib*, and *aac(3)-Iia*), β-lactams (*bla*CTX-M-15, *bla*TEM-1b, *bla*NDM-7, *bla*OXA-1, and *bla*VIM-1), fluoroquinolones (*qnrB20* and *qnrB2*), chloramphenicol (*catB3*), trimethoprim (*dfrA14*), tetracycline (*tetA*), and sulfonamides (*sul1-2*) (**Table 1**). Unexpectedly, compared with the 12 genomes, 8501 showed a narrower plasmid content, characterized by IncFIB(k), IncFII(k), IncN, and IncX3. Moreover, IncF-type plasmids turned out to be common in ST3564, unlike IncN and IncX3, detected in CR-Ec 8501 only.

### Genomic composition of pNDM-7

**Figure 3:**
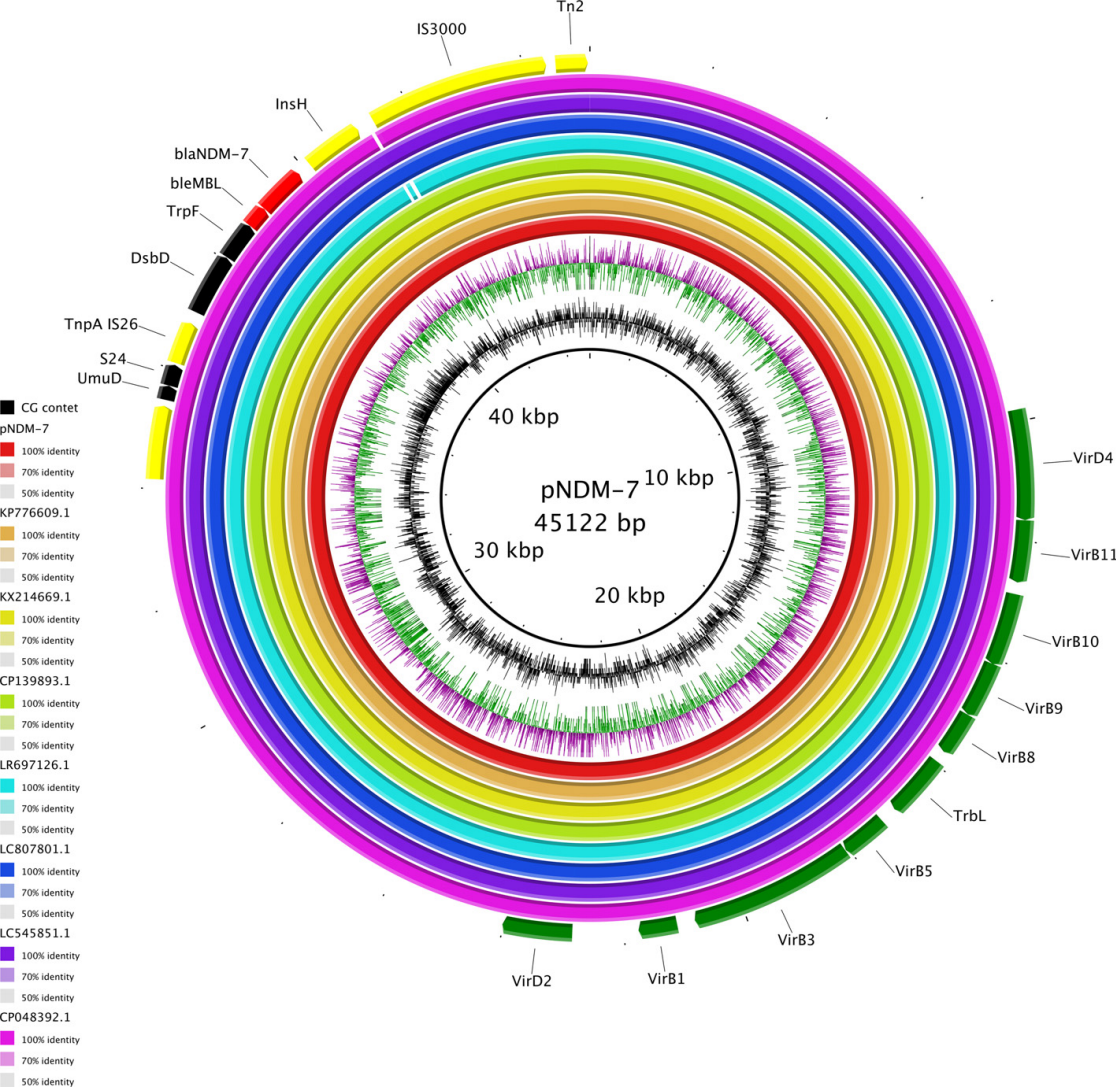
Circular map of pNDM-7 (red), pOM26-1 (KP776609.1, orange), pKW53T-NDM (KX214669.1, yellow), pNDM (CP139893.1, green), p14ARS_MMH0055-5 (LR697126.1, light blue), pRA123-NDM-7 (LC807801.1, blue), pEc-MW07_NDM (LC545851.1, purple), and pEc-MW07_NDM (CP048392.1, fuchsia). At the outer curved segments, red, yellow, green, and black correspond to antimicrobial resistance genes, mobile elements, transferability region, and other functions, respectively.

The carbapenemase gene *bla*NDM-7 was carried by an IncX3 plasmid of 45,122 bp. *bla*NDM-7 was included in an antimicrobial resistance island (ARI) of 11,686 bp composed as follows: Tn*3*-IS*Kox3*-*UmuD*-*S24*-*TnpA*-*DsbD*-*TrpF*-*ble*MBL-*bla*NDM-7-*InsH*-IS*3000*-Tn*2*. The entire pNDM-7, including ARI, showed complete identity with globally spread NDM-7-carrying plasmids, as pRA123-NDM-7 (LC807801.1) collected from a *K. pneumoniae* in a water sample in Bangladesh in 2021 and pOM26-1 (KP776609.1) from clinical *E. coli* in Oman in 2012 (query and identity 100% for all). High-level identities (query 99% and identity 100%) were pointed out for pKW53T-NDM (KX214669.1) from clinical *E. coli* in Oman in 2012, pNDM (CP139893.1) from clinical *E. coli* from China in 2021, p14ARS_MMH0055-5 (LR697126.1) from a *K. pneumoniae* collected in the UK, and pKPS01_p3 (CP048392.1) obtained from a clinical *K. pneumoniae* in Australia in 2016. Moreover, pNDM-7 showed high match (query 99% and identity 100%) with pEc-MW07 (LC545851.1), an NDM5-harboring IncX3 plasmid collected from a clinical *E. coli* in Malawi in 2017 (**Figure 3**). These data all together highlighted the conservative nature of *bla*NDM-like-harboring IncX3 plasmids, reflected in their successful circulation since at least 2012. Moreover, the *bla*NDM-7-harboring plasmid turned out to be transferable by conjugation.

## Discussion

Here we report the identification of three *E. coli* STs (ST401, ST355, and ST3564), usually found in animal reservoirs, harboring several carbapenemases (including the first Italian case of NDM-7), from patients admitted in critical wards, including the Intensive Care Unit.

*E. coli* is an important cause of morbidity and mortality in humans and animals worldwide. The fine ability of *E. coli* to adapt to different lifestyles and settings reflects its versatile nature and should be prioritized within the One Health framework involving humans, animals, and the environment. Although some *E. coli* STs have evolved to adapt to specific environments, others are found in both humans and animals, circulating through shared environments, food sources, or direct contact. In general, the STs identified in humans and animals differ for virulence and resistance determinants harbored, with the human-associated strains usually displaying a higher amount of resistance than virulent genes, and vice versa. Resistance to cephalosporins, fluoroquinolones, and aminoglycosides is reported as the most frequent phenotype in human *E. coli* isolates, while in animals, ESBL-producing *E. coli* isolates and resistance to penicillins, cephalosporins, and trimethoprim/sulfamethoxazole are more frequent (Giufrè et al., 2021). Resistance to carbapenems in *E. coli* remains uncommon in Europe in both humans and animals (EARS-Net, 2023; European Food Safety Authority (EFSA) and European Centre for Disease Prevention and Control (ECDC), 2024).

*E. coli* ST401 has been described to be associated with both poultry and aquatic environments (Vincent et al., 2010; Behruznia and Gordon, 2022; Constantinides et al., 2020). In particular, the poultry strains are often recognized as APEC and showed several virulence factors, primarily adhesins, invasins, and toxins (Kathayat et al., 2021), and fewer resistance determinants, with aminoglycoside, sulfonamide, and beta-lactam resistance genes as the mainly reported ones (Kathayat et al., 2021). The occurrence of clinically relevant carbapenemases, such as KPC and NDM types, in such strains possessing potential virulent features, is a worrying event. Here we report the first cases of *bla*KPC-3 and *bla*VIM-1 determinants harbored in ST401 *E. coli* strains from human sources. The different plasmidome content and the conjugation rate pointed out the ability of ST401 to acquire and fit several AMR traits. These data, together with a wide *armamentarium* of virulence genes, increase its infective potential in the human environment. These data suggest the ability of ST401 to fit in different environments.

*E. coli* ST355 is often identified among UPEC isolates, widely reported in the poultry environment and associated with fluoroquinolone resistance traits (Myrenås et al., 2018; Kaspersen et al., 2020). Based on the available metadata and on the literature, ST355 is rarely associated with beta-lactam resistance, while the *E. coli* 7926 isolated in this study present *bla*KPC-3 carbapenemase gene. These data highlighted the worrying entrance of successful carbapenemases, such as *bla*KPC-3, in *E. coli* ST355 with high virulence potential.

*E. coli* ST3564 belongs to the clonal complex ST23 and has not been characterized in the literature, yet. The only data reporting this ST are present in an annual report of the Danish surveillance program of AMR and consumption in humans and animals drafted in 2016 (Borck Høg et al., 2016). In that document was reported one isolate of ST3564 *E. coli* collected from a pig, harboring the *bla*CTX-M-1 gene. No carbapenemases have been detected, to date, in such an ST. Here, the 8501 strain, belonging to ST3564, presented, besides different β-lactamases, two metallo-β-lactamases, *bla*VIM-1 and *bla*NDM-7, here reported, to the best of our knowledge, for the first time in Italy. This allelic variant has already been reported in several regions, such as Japan (Mizuno et al., 2014), India (Rahman et al., 2014; Hammerum et al., 2015; Devanga Ragupathi et al., 2017; Paul et al., 2017; Ramakrishnan et al., 2021), China (Wang et al., 2016; Xu and He, 2019; Xia et al., 2022; Liu et al., 2024), Nepal (Shrestha et al., 2017), the Arabian Peninsula (Pál et al., 2017), Myanmar (Sugawara et al., 2019), Brazil (Rodrigues et al., 2021; de Oliveira Alves et al., 2022), Nigeria (Tickler et al., 2021), Fiji (Young-Sharma et al., 2024), and Europe in *Enterobacterales*, mostly *K. pneumoniae* and *E. coli*, and in different plasmids, mainly IncX3. In Europe, NDM-7 has been reported since 2013 and in Germany and France in two *E. coli* isolates belonging to ST599 and ST167, respectively (Göttig et al., 2013; Cuzon et al., 2013). In the same year, an interhospital outbreak of an ST437 NDM-7-producing *K. pneumoniae* clone occurred in Spain (Seara et al., 2015). In the following years, other sporadic cases of NDM-7 were described, mainly in Spain, in *E. coli* belonging to different STs (ST399, ST679, and ST167) and in plasmids of different types (IncX3 and IncX4) (Pérez-Moreno et al., 2016; Espinal et al., 2018; Pérez-Vázquez et al., 2019; Marí-Almirall et al., 2021). The fact that NDM-7-producing isolates are often found in strains from surveillance rectal swabs (van der Mee-Marquet et al., 2016; Solgi et al., 2017) highlights that more attention should be paid to the silent circulation of these clones, since they can asymptomatically colonize patients and be introduced into hospitals, thereby posing a major risk of infections in fragile patients, also considering that the *bla*NDM-7 gene is often driven by conjugative plasmids, such as the case of the IncX3 plasmid here reported, which can easily be transferred to other bacterial species. Moreover, *E. coli* 8501 was of serotype O8:H19, characteristic of porcine stx2-producing *E. coli* (Saupe et al., 2017), which is associated with mild disease and has occasionally caused hemolytic uremic syndrome in the Netherlands (Friesema et al., 2015). This report suggested a peculiar evolutionary turning point of ST3564, with a reduction in its virulence content and, on the other hand, an increase in its ability to acquire clinically relevant carbapenemases.

The above set of results confirms the thick bond between veterinary and human compartments in the diffusion of high-risk pathogens and emphasizes how the epidemiology of carbapenemases is constantly evolving, also highlighting the importance of genome-wide studies for better understanding of circulating clones and plasmids.

## Data Availability

The datasets presented in this study can be found in online repositories. The names of the repository/repositories and accession number(s) can be found in the article/**Supplementary Material**.

## Supplementary material

Supplementary Figure 1 linear visualization of 7926-pQiL and 7521-pQiL plasmids. Arrows show the direction of transcription of open reading frames. Green, transferability; blue, replication; yellow, mobile elements; red, antimicrobial resistance gene; fucsia, Type I restriction system; brown, partition system; grey, other functions. Light blue shading shows homologous segments with 100% sequence identity.
