# Supplementary material for: Polyclonal carbapenemase-producing Escherichia coli in Northern Italy: the emergence of NDM-7
Source: Front Cell Infect Microbiol. 2025 Mar 14;15:1519827. doi: 10.3389/fcimb.2025.1519827 (PMC11949906; doi:10.3389/fcimb.2025.1519827)
Supplement: Supplementary file 2 [file Table1.docx]

**Supplementary Table 1.** Antibiotic susceptibility profiles of the four *E. coli* strains studied. Interpretation according to EUCAST breakpoints are displayed in brackets. MIC values are reported in µg/ml.

| **Strain** | **AMC**  **S≤8**  **R>8** | **AMP**  **S≤8**  **R>8** | **ATM**  **S≤1**  **R>4** | **P/T**  **S≤8**  **R>8** | **FEP**  **S≤1**  **R>4** | **CTX**  **S≤1**  **R>2** | **CAZ**  **S≤1**  **R>4** | **CZA S≤8**  **R>8** | **C/T**  **S≤2**  **R>2** | **CFX S≤8**  **R>8** | **CRX**  **S≤1**  **R>2** | **CIP**  **S≤0.25 R>0.5** | **COL**  **S≤2 R>2** | **FOS***  **S≤8**  **R>8** | **ERT S≤0.5 R>0.5** | **IMP**  **S≤2**  **R>4** | **IMR**  **S≤2**  **R>2** | **MEM**  **S≤2**  **R>8** | **MEV**  **S≤8 R>8** | **AK**  **S≤8 R>8** | **CN**  **S≤2 R>2** | **TO**  **S≤2**  **R>2** | **SXT**  **S≤2**  **R>4** | |
| --- | --- | --- | --- | --- | --- | --- | --- | --- | --- | --- | --- | --- | --- | --- | --- | --- | --- | --- | --- | --- | --- | --- | --- | --- |
| 7521 | >32/2 (R) | >16 (R) | >32  (R) | >32/4 (R) | >16 (R) | 4  (R) | >16 (R) | <0,25 (S) | 8  (R) | > 16 (R) | > 4  (R) | <0.125  (S) | <05  (S) | 2  (S) | 1 (R) | 2 (S) | 0.25 (S) | 0.5 (S) | <0.06 (S) | <2 (S) | <1 (S) | <0.5  (S) | | ≤1/19 (S) |
| 7926 | >32/2 (R) | >16 (R) | >32  (R) | >32/4 (R) | 4  (R) | > 4 (R) | >16 (R) | <0,25 (S) | >8  (R) | > 16 (R) | > 4  (R) | <0.125  (S) | <0.5  (S) | 2  (S) | >2 (R) | 4 (R) | 2.05 (S) | 0.5 (S) | <0.06 (S) | <2 (S) | <1 (S) | 1  (S) | | ≤1/19 (S) |
| 8501 | >32/2 (R) | >8 (R) | >32  (R) | >16/4 (R) | >16 (R) | > 32 (R) | >16 (R) | >16  (R) | >8  (R) | >8 (R) | > 4  (R) | >1  (R) | <0.5  (S) | 8  (S) | >1 (R) | >8 (R) | >8 (R) | >16 (R) | >16 (R) | 16 (R) | >4 (R) | >4 (R) | | >4/76 (R) |
| 9008 | >32/2 (R) | >8 (R) | >32  (R) | >64/4 (R) | >16 (R) | > 32 (R) | >16 (R) | >16  (R) | >8  (R) | >8 (R) | > 4  (R) | 0.5  (I) | <0.5  (S) | 1  (S) | >1 (R) | 4  (R) | 4  (R) | >8  (R) | >8/8  (R) | <2 (S) | 4 (R) | >4 (R) | | >4/76 (R) |

AMC: Amoxicillin-clavulanate, AMP: Ampicillin, ATM: aztreonam; P/T: Piperaciilin/Tazobactam, FEP: Cefepime, CTX: Cefotaxime, CAZ: Ceftazidime, CZA: Ceftazidime/avibactam, C/T: Ceftolozane/tazobactam, CFX: Cefuroxime, CRX: Ceftriaxone; CIP: Ciprofloxacin, COL: Colistin; FOS: fosfomycin, *agar dilution; ETP: Ertapenem, IMP: Imipenem, IMR: Imipenem/relebactam; MEM: Meropenem, MEV: Meropenem/vaborbactam; AK: Amikacin, CN: Gentamicin, TO: Tobramycin; SXT: Trimethoprim/Sulfamethoxazole. S: Susceptible, standard dosing regimen; R: resistant.
