# Supplementary material for: Polyclonal carbapenemase-producing Escherichia coli in Northern Italy: the emergence of NDM-7
Source: Front Cell Infect Microbiol. 2025 Mar 14;15:1519827. doi: 10.3389/fcimb.2025.1519827 (PMC11949906; doi:10.3389/fcimb.2025.1519827)
Supplement: Supplementary file 3 [file Table2.docx]

| **Isolate** | **Short-read coverage** | **Long-read coverage** | **Assembly size** | **Number of contigs** | **N50** |
| --- | --- | --- | --- | --- | --- |
| 7521 | 77 | 5 | 5279468 | 25 | 922630 |
| 7926 | 60 | 7 | 5177490 | 15 | 3694097 |
| 8501 | 63 | 10 | 5130816 | 4 | 4855862 |
| 9008 | 60 | 11 | 5379275 | 17 | 3150856 |
|  |  |  |  |  |  |
| average | 65 | 8,25 | 5241762,25 | 15,25 | 3155861,25 |

**Supplementary Table 2.** General information on sequencing data
